# A Flexible Two-Sensor System for Temperature and Bending Angle Monitoring

**DOI:** 10.3390/ma14112962

**Published:** 2021-05-30

**Authors:** Yifeng Mu, Rou Feng, Qibei Gong, Yuxuan Liu, Xijun Jiang, Youfan Hu

**Affiliations:** 1 Hunan Institute of Advanced Sensing and Information Technology, Xiangtan University, Xiangtan 411105, China; 201721521305@smail.xtu.edu.cn (Y.M.); 201821521392@smail.xtu.edu.cn (R.F.); gqb_taoking@smail.xtu.edu.cn (Q.G.); 202021001531@smail.xtu.edu.cn (Y.L.); 202021001515@smail.xtu.edu.cn (X.J.); 2 Key Laboratory for the Physics and Chemistry of Nanodevices, Center for Carbon-Based Electronics, Frontiers Science Center for Nano-Optoelectronics, Department of Electronics, Peking University, Beijing 100871, China

**Keywords:** flexible sensors, temperature, angle, arthritis, wearable electronics

## Abstract

A wearable electronic system constructed with multiple sensors with different functions to obtain multidimensional information is essential for making accurate assessments of a person’s condition, which is especially beneficial for applications in the areas of health monitoring, clinical diagnosis, and therapy. In this work, using polyimide films as substrates and Pt as the constituent material of serpentine structures, flexible temperature and angle sensors were designed that can be attached to the surface of an object or the human body for monitoring purposes. In these sensors, changes in temperature and bending angle are converted into variations in resistance through thermal resistance and strain effects with a sensitivity of 0.00204/°C for temperatures in the range of 25 to 100 °C and a sensitivity of 0.00015/° for bending angles in the range of 0° to 150°. With an appropriate layout design, two sensors were integrated to measure temperature and bending angles simultaneously in order to obtain decoupled, compensated, and more accurate information of temperature and angle. Finally, the system was tested by being attached to the surface of a knee joint, demonstrating its application potential in disease diagnosis, such as in arthritis assessment.

## 1. Introduction

Increasing numbers of wearable electronics have come into our daily lives and have greatly changed the way we interact with the physical world. Generally, flexibility [1], stretchability [2,3], biocompatibility [4,5], and foldability [6] are desirable in wearable electronics [7,8] due to the fact that a comfortable, stable, and safe interface needs to be established between the device and the biological surface; this cannot be achieved using existing electronic devices, which are usually stiff and fragile [9]. As a new form of electronics, wearable devices are expected to play an important role in health/medical monitoring [10,11], big data acquisition [12,13], soft robots [14,15,16], and so forth. In particular, with the rapid increase in demands to improve people’s quality of life and the growing awareness of medical conditions, various flexible sensors that can be attached to the skin to record physiological signals have been developed for health/medical monitoring purposes. Information such as skin temperature [17,18], heart rate [19,20], respiratory rate [21,22], blood pressure [23,24], sweat metabolite concentrations [25,26], electrical activity of the heart [27,28], electrical activity in the brain [29,30], body position and movement [31,32], and so forth can be obtained. For example, in a previous study, heart rate could be recorded via wrist pulses by monitoring the changes in a micropatterned ultrathin-film-based resistor attached to the wrist, which was constructed with a mixture of polydimethylsiloxane (PDMS) and single-walled carbon nanotubes [19]. The recorded pulses present a clear percussion wave (P-wave), tidal wave (T-wave), valley, and diastolic wave (D-wave), which can provide very useful medical information. In another case, human sweat was sampled via a wearable integrated sensor array, and sweat metabolites (such as glucose and lactate) and electrolytes (such as sodium and potassium ions) could be monitored to enable the real-time assessment of the exercise intensity of the human body [33].

Among these signals that can be obtained from the human body, the variation pattern of skin temperature often provides crucial information for diagnosing vascular diseases, assessing physical activity, and monitoring infection/inflammation conditions [34,35,36]. For example, previous investigations have shown that, for normal control subjects, wounds with a diagnosis of infection or signs of inflammation evidenced a relative temperature elevation of 1.1 to 1.2 °C compared with those without the condition of infection or inflammation [36]. Meanwhile, as temperature is a common influencing factor in various sensors, temperature information is also necessary in order to carry out the calibration of other sensors in an integrated system [33,37]. Recently, body position and movement monitoring has been afforded increasing attention due to its important role in biomechanical measurements of motion dynamics and sports medicine and the medical assessment of diseases related to muscles and joints and so forth [38,39,40,41,42] For instance, data recorded by wearable triaxial accelerometers and gyroscopes were used to classify tremor and bradykinesia in patients with Parkinson’s disease [38]. From these progresses, we can also see that obtaining multidimensional information is desirable. Generally, there are two approaches to achieve this goal: one is using a single sensor to monitor multiple stimuli, which requires complex subsequent signal processing in order to decouple the complex and mixed information [43,44,45]; the other method is to use multiple sensors that only respond to specific stimuli to obtain decoupled information directly [46,47]. In this approach, the fabrication process is generally complex, and a sophisticated mechanical design of the integrated system is needed. Therefore, with the development of wearable technology, new requirements have arisen in order to progress to the next stage of development, including but not limited to the integration of multiple sensors to obtain multidimensional information for more accurate situation estimation, independent sensor responses to enable an easy and quick signal analysis process, low-cost fabrication with the capacity to be scaled up, and good mechanical durability for long-term usage. Here, we propose a feasible method to construct a flexible two-sensor system that can be attached to the surface of an object or the human body for the monitoring of temperature and bending angle. By properly designing the relative positions, orientations, and footprints of two serpentine-structured resistive sensors, the temperature sensor was isolated from mechanical deformation to provide an independent measurement of temperature, while deformation was maximized in the strain sensor, and more accurate information of bending angle could be obtained after compensation for temperature. This approach is low cost and has the capacity to be scaled up. The ability of the system to record the movement and local skin temperature of the knee joint has been demonstrated, revealing its application potential in physical activity monitoring and disease (e.g., in arthritis diagnosis).

## 2. Materials and Methods

### 2.1. Device Fabrication

**Figure 1:**
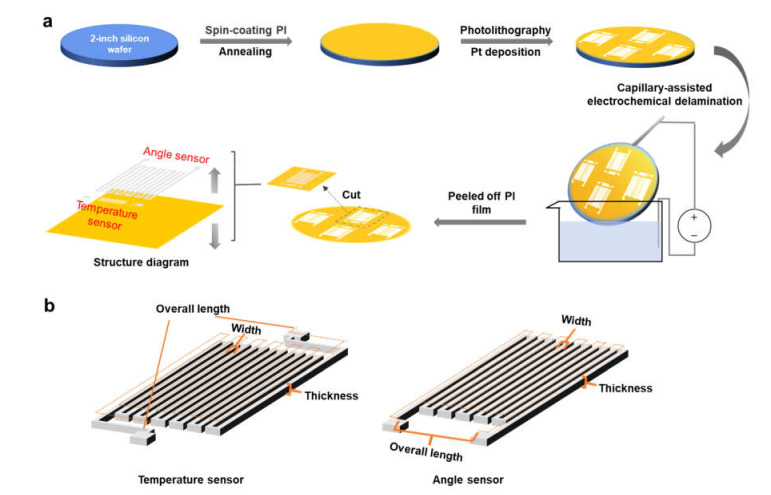
Schematic illustration of (**a**) the fabrication procedures and (**b**) a temperature sensor and an angle sensor marked with design parameters.

Figure 1a shows a schematic illustration of the fabrication procedure used to make the devices. A 2-inch silicon wafer was utilized as the supporting substrate during the fabrication process; it was purchased from Tianjin SEMI Tech. Res. Inst. and had a resistivity of 0.009 Ω cm to ensure a high conductivity and efficient electrochemical reactions in the following steps. First, the silicon wafer was cleaned by rinsing it in deionized water, acetone, and isopropanol in sequence; then, poly (pyllellic dianhydride co-4,4′-oxydianiline), amic acid solution was spin-coated on it at a rotational speed of 4000 rpm and annealed at 150 °C for 5 min and 250 °C for 1 h on a hot plate to form a 5 μm-thick polyimide (PI) film. Compared with other flexible substrates, such as polyethylene terephthalate and polyethylene naphthalate, PI has the advantages of having high heat resistance, low thermal expansion, low surface roughness, and excellent mechanical strength at several micrometers, which are beneficial properties for microfabrication and can help to achieve a very thin system to realize conformal contact with curved surfaces. Then, photolithography was carried out to define the designed patterns on the PI film, Pt was deposited on the substrate to construct the desired sensors, and a thin layer of Ti (50 nm in a temperature sensor, 20 nm in an angle sensor) was first deposited under the Pt to promote the adhesion of Pt on the PI film. Finally, the PI film was peeled off from the silicon wafer using the capillary-assisted electrochemical delamination method [48] and cut into different pieces, with each one containing a system. In the delamination process, the PI film along the edge of the silicon wafer was first trimmed to expose the underlying silicon wafer. Then, the silicon wafer was tilted, and its lower exposed edge was placed in contact with a NaCl electrolyte solution. A voltage of 5 V was applied between the silicon wafer and the electrolyte solution to trigger an electrochemical reaction at the silicon wafer surface and generate gaps at the interface between the PI film and the silicon wafer. With the aid of the capillary forces introduced via these gaps, the electrolyte solution climbed along the interface, and an electrochemical reaction took place throughout the silicon wafer surface. Finally, the PI film with sensor systems on its top surface was delaminated from the silicon wafer gently without suffering any appreciable mechanical or chemical damage. The bottom left of Figure 1 shows the structure diagram for the two-sensor system with separate layers for the device and the PI substrate, and a schematic diagram of an obtained device marked with design parameters is shown in Figure 1b.

### 2.2. Calculation Method for Bending Angle

**Figure 2:**
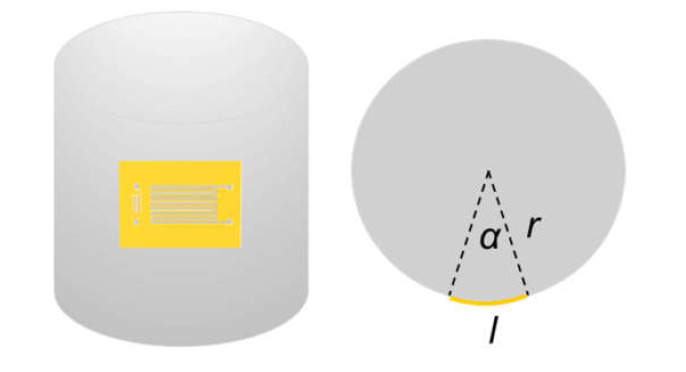
Schematic diagram showing how the bending angle is calculated.

Figure 2 schematically shows how the bending angle was calculated when the device was attached to the cylinder surface. In this figure, *l* is the length of the PI film along the deformation direction, *r* is the radius of the acrylic cylindrical rod, and *α* is the bending angle of the device, which can be estimated as α=180l/πr.

## 3. Results and Discussion

### 3.1. Temperature Sensor

**Figure 3:**
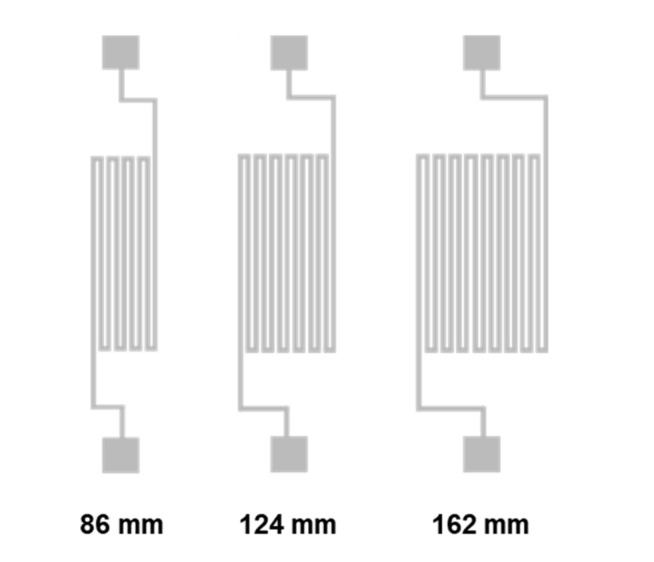
Design diagrams of 86, 124, and 162 mm-long devices.

Temperature sensors were constructed with serpentine-shaped Pt wires based on the thermal resistance effect. Pt is the most common metal used for resistance temperature sensors because of its relative high-temperature coefficient of resistance, excellent corrosion resistance, excellent long-term stability, and wide working range. A serpentine shape was adopted to obtain a sufficient resistance value within a small footprint for the metal wire. To find an optimized structure, three serpentine Pt wires designed with the same width of 100 μm, the same thickness of 200 nm, and different overall lengths of 86, 124, and 162 mm were tested. The design diagrams of these devices are shown in Figure 3.

**Figure 4:**
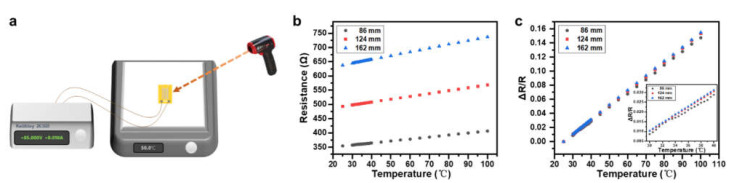
(**a**) Schematic diagram of the experiment setup for the temperature-sensing characterization of the device. (**b**) Resistance change in response to temperature of three temperature sensors with different lengths of wires. (**c**) Relative resistance change as a function of the temperature of these three sensors. Inset: relative resistance changes as a function of temperature in the temperature range from 30 to 40 °C.

During the assessment, these sensors were placed on a hot plate surface and elevated to different temperatures in the range of 25 to 100 °C. A commercial infrared thermometer (UT300S, UNI-TREND TECHNOLOGY, Dongguan, China, with an accuracy of ±2% at 23 ± 2 °C and a resolution of 0.1 °C) was used to monitor the surface temperature of the sensor, as shown schematically in Figure 4a. The resistances of the Pt wires were measured by a Keithley 2636B System SourceMeter^®^ (Keithley Instruments, Cleveland, USA). The resistance can be expressed as *R* = *ρL*/*S*, where *ρ* is the resistivity, *L* is the overall length, and *S* is the cross-sectional area of the metal wire. Among these parameters, *L* and *S* are geometric factors and are almost unaffected by temperature, while *ρ* is a temperature parameter. In a relatively narrow temperature range, *ρ* may be linearly approximated as, ${\rho =\rho }_{0}{(1+\alpha (T-T}_{0}))$, where $\rho_{0}$ is the resistivity at the reference temperature $T_{0}$ and *α* is the temperature coefficient of resistivity. As the *α* of Pt is constant over the tested temperature range, the measured resistances of these three devices, as shown in Figure 4b, reveal linear changes with temperature at the same rate, and the separation of these three recorded lines reveals the original resistance difference in these metal wires due to their different overall lengths.

The changes of relative resistance, $\frac{\Delta R}{R}=\frac{{R-R}_{0}}{R_{0}}$, with the temperature of these three devices were calculated as shown in Figure 4c. The sensitivity, $S=\frac{\delta \frac{\Delta R}{R}}{\delta T}$, was estimated to be 0.00198/°C, 0.00204/°C, and 0.00207/°C from these curves for the 86, 124, and 162 mm-long devices, respectively, which is almost double the number found by a previous report that was also based on serpentine metal wires but with printed Ag nanoparticles [49] and outperforms the results based on Ti/Pt/Cr/Au thin film [50] and patterned single-layer graphene [51]. The better performance achieved here may be the result of a better quality of the Pt thin-film fabricated via sputtering. The inset in Figure 4c shows the relative resistance change in the temperature range of 30 to 40 °C, which is especially meaningful for monitoring the skin temperature of the human body. The curve of the 86 mm-long sensor shows a degraded linearity compared with the other two curves in the temperature range of 30 to 40 °C, while the footprint of the 162 mm-long device was a little larger, which may introduce other factors; here, this includes deformation, which could interfere with the response of the sensor to temperature. As a result, a temperature sensor with a 124 mm-long trace was selected as the temperature-sensing component for further integration.

### 3.2. Angle Sensor

**Figure 5:**
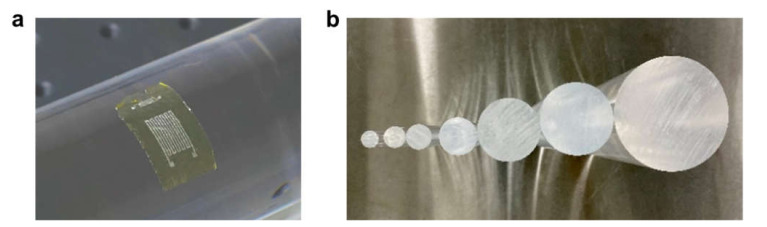
(**a**) A certain bending angle is introduced in the device by attaching the device to an acrylic cylinder rod. (**b**) Acrylic cylinder rods with increased radii of 11, 13, 17, 23, 34, 45, and 67 mm.

Angle sensors are constructed based on the strain effect. According to $R=\frac{\rho L}{A}=\frac{\rho }{V}L^{2}$, where *V* is the volume of the wire that remains constant as *ρ* does during deformation, the serpentine structure is also adopted here to obtain a longer length, *L*, of the wire in a limited area to achieve higher sensitivity. To introduce bending deformations uniformly to the sensor, the device was attached to the surface of an acrylic cylinder rod after a thin layer of water was sprayed on the cylinder surface, as shown in Figure 5a, and a series of acrylic cylinder rods with different radii were used to introduce different bending angles, as shown in the inset in Figure 5b. One aspect that should be mentioned here is that the orientation of the longitudinal section of the metal wire was aligned with the direction of deformation, as exhibited in Figure 5a, to maximize the strain experienced by the sensor. 

**Figure 6:**
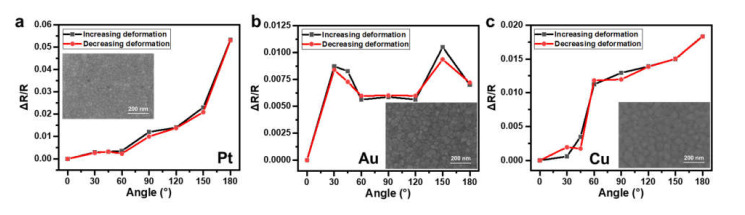
Relative resistance changes in response to the angles of sensors constructed with (**a**) Pt, (**b**) Au, and (**c**) Cu. Inset: SEM images of the surface morphology of corresponding metal wires.

Sensors constructed with Pt, Au, and Cu serpentine wires of the same geometry, which all had a width of 350 μm, a thickness of 80 nm, and an overall length of 212 mm, were tested by measuring their resistance changes when attaching them to different acrylic cylinder rods at a temperature of 25 °C. For every device, one curve was recorded while the bending angles were increased (i.e., increasing deformation), and the other one was recorded while the bending angles were decreased (i.e., decreasing deformation). These are shown in Figure 6a–c for Pt-, Au-, and Cu-based sensors, respectively. Comparing the response characteristics of these devices, the device constructed with Pt wires showed a better sensitivity, linearity, and repeatability. The scanning electron microscope (SEM) images of the surface morphology of these wires shown in the inset in Figure 6 reveals that the grain size in the Pt wire was about 30–50 nm, which was much smaller than that in the Au and Cu wires (100–120 nm). These grain size differences may have resulted in different morphology changes under deformation, and thus a different sensitivity, linearity, and repeatability of the constructed sensors. It should be mentioned that the repeatability in all devices was good, which means that the processes of attaching and detaching the device to different cylinders to introduce different bending angles did not influence the device performance. The obtained sensitivities of the devices made of Pt were 0.00015/° (average of two curves) with a linearity (defined by the coefficient of determination) of 0.9296 in the range of 0° to 150° and 0.00101/° (average of two curves) in the range of 150° to 180°, which is comparable to previous results reported for angle sensors [52,53].

**Figure 7:**
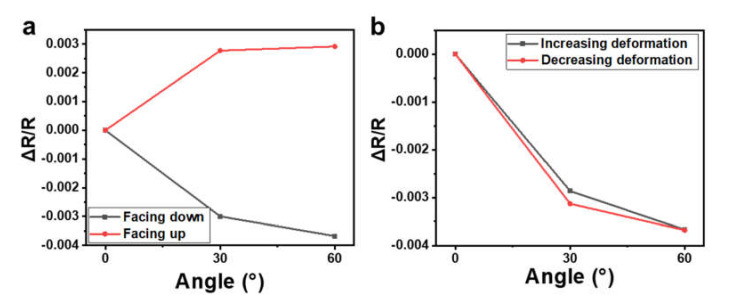
(**a**) Relative resistance changes in response to angles when the sensor was tested facing down and facing up. (**b**) Relative resistance changes in response to the angles of the sensor tested facing down while the bending angles were increased and decreased.

As the Pt wires responded to changes in angle because of the produced strain during this deformation, the response of the device varied depending on the mounting methods of the device on a curved surface (facing up or down), because that may have resulted in tensile strain or compressive strain at the wire. Figure 7a reveals that the device showed good responsibility to changes in angle when tested while attached to the cylinder both facing up and facing down, and good reproducibility was also shown when tested facing down (Figure 7b), similar to when tested facing up (Figure 5a).

**Figure 8:**
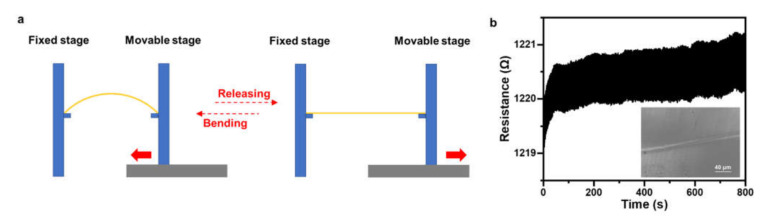
(**a**) Schematic diagram of the experimental setup for the repeated bending test. (**b**) Resistance recorded for an angle sensor during 800 cycles of repeated bending. Inset: SEM image of the surface morphology of the Pt wire after bending test.

The mechanical stability of the angle sensor was further verified by a repeated bending test. The device was mounted between two sample stages, as shown schematically in Figure 8a. One stage was fixed, and the other one was movable and controlled by a motorized linear stage (M-ILS150CC, Newport, Irvine, USA) to introduce a bending curvature of 0.02 mm^−1^ in the device. The device was bent for 800 cycles at a frequency of one bend per second, with the results shown in Figure 8b. This reveals that although there was a slight increase in resistance during the first 50 bending cycles, the relative resistance change was no more than 0.19%, which demonstrates an excellent mechanical stability. We checked the surface morphology of the Pt wire after the repeated bending test. Microcracks were generated in the wire, as shown in the inset in Figure 8b. We attribute the slightly increased resistance during the first 50 bending cycles to the generation of these microcracks. After that, the resistance of the device was very stable with a fluctuation of less than 0.5 Ω.

### 3.3. The Two-Sensor System and Its Applications

**Figure 9:**
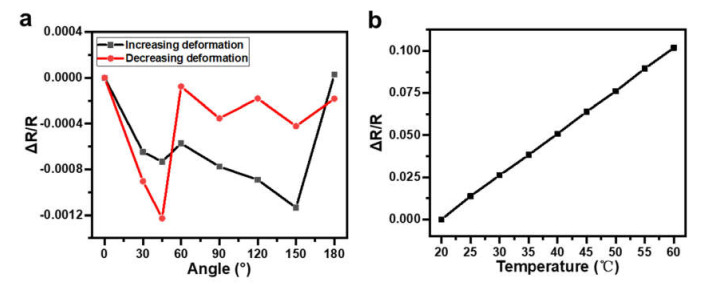
(**a**) Response of the temperature sensor to the change in angle. (**b**) Response of an angle sensor to the change in temperature.

When constructing a multiple-sensor system, obtaining the desired information using independent sensor responses to different stimulations is preferred. Here, in order to obtain decoupled temperature and angle information, the layout of the integration of the temperature sensor and angle sensor was designed as shown schematically in the bottom left of Figure 1 and in the optical photograph shown in Figure 5a. The angle sensor occupied a large area, with the longitudinal section of the serpentine wire oriented to align with the direction of the deformation, while the temperature sensor was constrained to a small footprint with the longitudinal section perpendicular to the deformation direction to obtain minimum interference from mechanical disturbance. To verify this design, Figure 9a shows the relative resistance change in the temperature sensor in response to different angles when attached to the surfaces of different acrylic cylinder rods with different radii at a temperature of 25 °C, revealing a fluctuation of no more than 0.123%. This indicates that the temperature sensor was nonsensitive to angle changes with this proper layout and that the temperature could be measured independently. However, in the current design, the angle sensor cannot be insulated from the surrounding temperature change. Figure 9b shows the response of the angle sensor to temperature when no bending deformation was introduced, which shows a very good linearity. Thus, combined with the temperature information provided by the temperature sensor, the angle information could be calibrated with temperature compensation, which was more accurate. The calibration process was conducted as follows: First, the temperature was measured by the temperature sensor; then, the resistance value of the angle sensor under the corresponding temperature without bending could be obtained by referring to Figure 9b. After that, this resistance value was used as the baseline to be deducted from the measured resistance of the angle sensor when it was bent. Finally, the $\frac{\Delta R}{R}$ of the angle sensor with calibration was used to deduce the bending angle by referring to Figure 6a.

**Figure 10:**
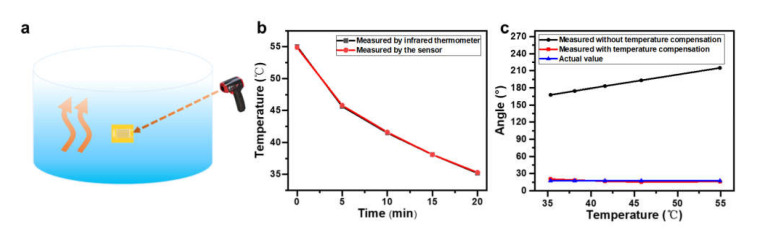
(**a**) Schematic diagram of a two-sensor system attached to the surface of a cylindrical container filled with hot water. (**b**) Temperature change over time measured on the container surface by an infrared thermometer and temperature sensor. (**c**) Angle measured on the container surface at different temperatures with and without temperature compensations. The actual angle value of the container is also plotted as a reference.

In order to check the feasibility of the two-sensor system, it was first attached to the outer wall of a polypropylene cylinder vessel containing hot water at 60 °C, and the temperature at the surface of the temperature sensor was monitored by a commercial infrared thermometer, as shown schematically in Figure 10a. The resistances of the temperature and angle sensors were recorded every 5 min. Referring to Figure 4c, the temperature measured by the sensor can be deduced as shown in Figure 10b, which reveals an excellent fidelity to the data measured by the infrared thermometer and an average relative error of less than 0.25%. Figure 10c shows the angle value measured by the angle sensor before and after the calibration with temperature compensation. As revealed by the results shown in Figure 4c and Figure 6a, Pt wires showed a more significant response to temperature than to deformation. Therefore, it is remarkable that the accuracy of angle measurement was greatly improved after temperature compensation with the relative errors reduced from 1128% to less than 10%.

**Figure 11:**
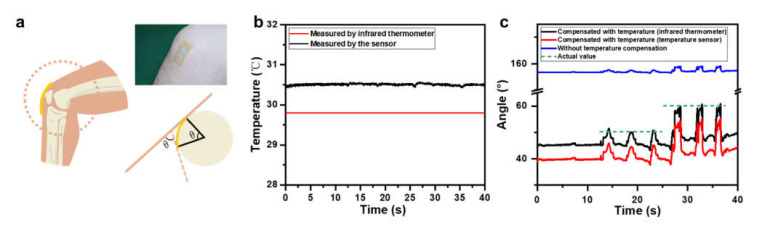
(**a**) Schematic diagram and optical photograph of a two-sensor system attached to the surface of a knee. (**b**) Temperature measured by an infrared thermometer and by a temperature sensor when attached to the knee. (**c**) Angle measured with and without temperature compensation during joint movement. The temperature used for compensation was measured by either an infrared thermometer or the temperature sensor. The actual value is also plotted as a reference.

Simultaneously recording temperature and angle information can be valuable and provide quantitative references for disease diagnosis and progression monitoring. For example, monitoring joint temperature and its range of motion can be used to evaluate the development and degree of rehabilitation after treatments for arthritis, which is a leading cause of disability. A simplified cross-sectional sketch of the knee joint with a two-sensor system attached to its surface is shown in Figure 11a. The two-sensor system was attached to the knee by using a liquid bandage (Hynaut) as the adhesive. First, the liquid bandage was sprayed on the skin surface. The liquid quickly formed a viscous film in 10–30 s. Then, the PI film with sensors was attached. The angle measured by the angle sensor, marked as *θ*, could be used to evaluate the knee flexion. First, the temperature at the knee surface was measured by the temperature sensor and by the commercial infrared thermometer, as shown in Figure 11b. The fidelity was degraded compared with the former test due to the deformations introduced into the temperature sensor by the complex surface of the joints. Even so, this deviation was still below 0.6 °C, and the fluctuation of the measured temperature was less than 0.148 °C. Figure 8b shows the angles recorded during joint movement before and after temperature compensation; Figure 11c shows the angles recorded during joint movement before and after temperature compensation, in which different degrees of knee flexion are recorded. The actual bending angles of the two statuses of knee flexion were 50.25° and 60.15°, recorded by an electronic digital angle ruler (SYJDC200, Syntek, Hangzhou, China). These two angles were 157.69° and 159.11° measured by our angle sensor without compensation and were 45.94° and 55.35° compensated with the temperature measured by the temperature sensor. The average error of these two angles reduced from 189.2% to 8.3%. If we were to use the temperature measured by the infrared thermometer to perform the compensation, the bending angle would be 51.44° and 60.85°, and the average error would be further reduced to 1.8%. Thus, improving the fidelity of temperature measurement would further increase the accuracy of the system. The determination and quantitation of different degrees of knee flexion demonstrate the system’s application potentials for assisting in arthritis assessment. 

## 4. Conclusions

In summary, we present a flexible two-sensor system constructed with serpentine-shaped Pt wires to record the temperature and bending angle simultaneously. With a rational design featuring appropriate relative positions, different footprints and orientations of the two sensors, decoupled, compensated, and more accurate information of temperature and angle can be obtained from the surface to which they are attached. The demonstration of the system in operation on a knee joint shows its feasibility to capture information from the body via a combination of easy fabrication and rational mechanical design. Introducing stretchability into the system will further improve the comfort of wearers and be conducive to long-term monitoring. This can be done in the next step by attaching the whole system to a prestreched silicone substrate to introduce a wave structure into the system, which can result in a stretchability up to the prestrain of the silicone substrate [4], or by cutting the PI substrate into some special patterns and encapsulating it with rubber [54,55]. Furthermore, incorporating more sensors into the system with a well-designed system structure to provide more complete information would greatly elevate the capacity of the system to be used in practical applications in the areas of health monitoring, clinical diagnosis, and therapy.

## Data Availability

The data presented in this study are available on request from the corresponding author.
